# Pituitary Adenylate Cyclase-Activating Peptide (PACAP)-Glutamate Co-transmission Drives Circadian Phase-Advancing Responses to Intrinsically Photosensitive Retinal Ganglion Cell Projections by Suprachiasmatic Nucleus

**DOI:** 10.3389/fnins.2019.01281

**Published:** 2019-12-06

**Authors:** Peder T. Lindberg, Jennifer W. Mitchell, Penny W. Burgoon, Christian Beaulé, Eberhard Weihe, Martin K.-H. Schäfer, Lee E. Eiden, Sunny Z. Jiang, Martha U. Gillette

**Affiliations:** ^1^Neuroscience Program, University of Illinois at Urbana-Champaign, Urbana, IL, United States; ^2^Department of Cell and Developmental Biology, University of Illinois at Urbana-Champaign, Urbana, IL, United States; ^3^Institute of Anatomy and Cell Biology and Center of Mind, Brain and Behaviour, University of Marburg, Marburg, Germany; ^4^Section on Molecular Neuroscience, Laboratory of Cellular and Molecular Regulation, National Institute of Mental Health, Bethesda, MD, United States

**Keywords:** PACAP, glutamate, suprachiasmatic nucleus, circadian rhythm, phase advance

## Abstract

Results from a variety of sources indicate a role for pituitary adenylate cyclase-activating polypeptide (PACAP) in light/glutamate-induced phase resetting of the circadian clock mediated by the retinohypothalamic tract (RHT). Attempts to block or remove PACAP’s contribution to clock-resetting have generated phenotypes that differ in their responses to light or glutamate. For example, previous studies of circadian behaviors found that period-maintenance and early-night phase delays are intact in PACAP-null mice, yet there is a consistent deficit in behavioral phase-resetting to light stimulation in the late night. Here we report rodent stimulus–response characteristics of PACAP release from the RHT, and map these to responses of the suprachiasmatic nucleus (SCN) in intact and PACAP-deficient mouse hypothalamus with regard to phase-resetting. SCN of PACAP-null mice exhibit normal circadian rhythms in neuronal activity, but are “blind” to glutamate stimulating phase-advance responses in late night, although not in early night, consistent with previously reported selective lack of late-night light behavioral responsiveness of these mice. Induction of CREB phosphorylation, a hallmark of the light/glutamate response of the SCN, also is absent in SCN-containing *ex vivo* slices from PACAP-deficient mouse hypothalamus. PACAP replacement to the SCN of PACAP-null mice restored wild-type phase-shifting of firing-rate patterns in response to glutamate applied to the SCN in late night. Likewise, *ex vivo* SCN of wild-type mice post-orbital enucleation are unresponsive to glutamate unless PACAP also is restored. Furthermore, we demonstrate that the period of efficacy of PACAP at SCN nerve terminals corresponds to waxing of PACAP mRNA expression in ipRGCs during the night, and waning during the day. These results validate the use of PACAP-deficient mice in defining the role and specificity of PACAP as a co-transmitter with glutamate in ipRGC-RHT projections to SCN in phase advancing the SCN circadian rhythm in late night.

## Introduction

Internal circadian clocks must be both self-sustaining and flexible if they are to be useful in organizing behavior and physiology. Despite discoveries regarding peripheral oscillators, the circadian clock in the SCN is still considered one of the few sets of mammalian cells that maintains its own circadian rhythm relative to the external environment. It does this by responding to external time cues, the most important of which for mammals is light. This combination of internal rhythm generation and sensitivity to change makes the SCN an intriguing model of intrinsic mammalian brain function, and its adaptation to the environment.

In addition to the actions of glutamate (Ding et al., 1994) there is some evidence that PACAP plays a role in mammalian clock resetting. PACAP is a member of the secretin/glucagon/vasoactive intestinal peptide (VIP) family originally isolated from ovine hypothalamus (Miyata et al., 1989). Since its discovery only 18 years ago, several roles have been established for PACAP, including mediation of neurotransmitter release, vasodilation, bronchodilation, mediation of intestinal activity, increase of insulin and histamine secretion, and cell multiplication and differentiation (reviewed in Vaudry et al., 2000). PACAP has been co-localized with glutamate in the terminals of the RHT on the SCN (Hannibal et al., 1997, 2000, 2001b) and has been found to shift the clock under certain conditions (Hannibal et al., 1997). PACAP has also been shown to be associated with melanopsin, one of photopigments of the retina necessary for clock entrainment (Panda et al., 2002; Ruby et al., 2002).

Exogenous application of PACAP has been more controversial, alternately causing delays (Piggins et al., 2001), light-like shifts (Harrington et al., 1999), or no effect alone but modification of light or glutamate-induced shifts (Chen et al., 1999; Tischkau et al., 2000). In PACAP- and PACAP receptor-deficient mice, designed to test PACAP’s circadian role, altered shifting that is not clearly consistent either with the previous pharmacological manipulations of PACAP in the SCN or with each other has been reported (Chen et al., 1999; Hannibal et al., 2001a; Kawaguchi et al., 2003; Colwell et al., 2004).

Another strain of mice was generated in 2002 that does not express detectable levels of PACAP (Hamelink et al., 2002). Like the Colwell model (Colwell et al., 2004), it was generated on the background of the C57BL6 mouse, but it eliminated only the PACAP-coding sequence, not the PRP sequence found in exon 4 (Okazaki et al., 1995). Thus, this model may represent a more specific lesion of the PACAP peptide sequence and was generated on a background strain most easily comparable to other mice that have been described with respect to circadian phenotype (Buchanan, 2002; Abbott, 2005) including the PAC1-null mice (Hannibal et al., 2001a). Examination of this strain of mice was designed to complement previous experiments on PACAP transgenic mice and to extend findings into a new modality. By replicating some previous experiments we hope to shed light on an earlier controversy – the discrepancy of shifting in systems with a disruption of PACAP signaling. By introducing another level of examination – the brain-slice preparation – we sought to further test the reliability of behavioral results and to create another method to answer signaling questions at the level of the SCN.

Each examination of PACAP-deficient compared to wild-type mice tested the effect of light on a background of constant conditions. While each strain of mouse showed significant differences in light-induced phase resetting compared to wild-type mice, PACAP-receptor null mice differed in phenotype from PACAP null mice (Hannibal et al., 2001a; Kawaguchi et al., 2003; Colwell et al., 2004). The alternate lesions of a peptide and its receptor, with complementary results, are basic criteria that establish each animal as a preferred organism with which to study that peptide signaling system. This approach was championed by Harmar et al. (2002) for PACAP/VIP signaling in SCN (Shen et al., 2000; Cutler et al., 2003; Aton et al., 2005), and is extended here. We have previously reported that light exposure to mice specifically deficient in PACAP, but not lacking PACAP-related peptide (PrP) expression (Hamelink et al., 2002) results in shifts reminiscent of the original PACAP receptor null mice generated by Hannibal et al. (2001a), (Beaule et al., 2009).

We here examine SCN from PACAP-null, compared to wild-type mice, in a brain slice preparation, to extend our previous examination of the phase-shifting phenotype of PACAP-deficient mice *in vivo* to the mechanisms of PACAP action at the retinohypothalamic synapse in the SCN itself. In this slice preparation, the SCN expresses a peak in spontaneous firing rate during midday, between CT6 and 7. This peak recurs ∼24 h later, at the same circadian time (Prosser and Gillette, 1989), and is shifted by a variety of stimuli in a manner consistent with the behavioral responses seen in the intact animal (Ding et al., 1994; Chen et al., 1999; Harrington et al., 1999; Tischkau et al., 2000; Buchanan and Gillette, 2005). Shifts represent a change in phase of the SCN, since peaks after a treatment recur ∼24 h after the first (shifted) peak (Gillette and Prosser, 1988; Hannibal et al., 1997). This preparation applied to PACAP transgenic animals thus has the power to study altered physiology in the same ways that it has previously been used to study relatively intact physiology and response to circadian signals of change. In the past, this preparation has been used to attempt to study a role of PACAP in circadian shifting (Chen et al., 1999). In PACAP-null mice, this preparation has the advantage of assured elimination of PACAP signaling. It is first important, however, to run appropriate controls for a system that has been otherwise lesioned over the course of the animals’ development.

Since PACAP-null mice maintain an endogenous peak in spontaneous SCN firing-rate frequency that is similar to wild-type animals, it is possible to compare their response to glutamate applied directly to the SCN. Importantly, this preparation also allows the controlled replacement of PACAP into a system with otherwise undetectable levels of the peptide (Hamelink et al., 2002). This replacement makes possible an important control for transgenic animals that has not been performed in previous models, and suggests means of further characterizing the role of PACAP in the SCN.

## Materials and Methods

### Materials

Peptides were purchased from AnaSpec (Fremont, CA, United States) unless otherwise noted. Antibodies were all obtained from commercial sources and these are indicated throughout the section “Results.”

### Animals

All manipulations were performed in accordance with the guidelines of the Institutional Animal Care and Use Committee and the Division of Animal Resources at the University of Illinois at Urbana–Champaign. Homozygote PACAP-null mice were derived as described previously (Hamelink et al., 2002) and bred onto a C57BL/6N background through 12 successive back-crossings (Hamelink et al., 2002). C57BL/6N mice were obtained from the same commercial source as the C57Bl/6N mice used to establish the PACAP null backcross line (Charles River).

Hypothalamic tissue explants with optic nerves intact were obtained from rats (Long-Evans) and mice (C57Bl/6) using previously described methods (Burgoon et al., 2004).

### Enucleation Surgery and Circadian Timing

Mice 6–24 weeks old were housed under 12-h light:12-h dark (LD) cycles and provided food and water *ad libitum*. Zeitgeber time (ZT) is determined from the animal’s LD cycle, with time of lights-on designated ZT 0. Because brain slices are maintained in constant conditions where the clock functions with a period of ∼24-h without external time cues, the time of lights-on in the donor colony is designated as circadian time 0 (CT 0). Thus, subjective day (CT 0–12) corresponds to the light portion of the donor’s former lighting schedule; subjective night (CT 12–24) corresponds to the dark portion of the donor’s cycle. For animals in constant darkness (when referred to in comparison to the studies performed here), onset of activity – designated by convention as CT12 – is used to determine period and indicate treatment times. Period is estimated by averaging the time between activity onsets for the 5 days prior to treatment. Treatment times are determined in circadian time to compensate for periods different than 24 h. For enucleated animals, onset of wheel-running activity – designated by convention as CT 12 – is used to determine period and treatment times (Beaule et al., 2009).

Bilateral orbital enucleation was performed on 8-week-old male C57Bl/6J mice (Jackson Labs, Bar Harbor, ME, United States) under ketamine, medetomidine anesthesia, by severing the optic nerve, muscle and other connective tissues, and removing the eyes. The eye cavity was packed with sterile Gelfoam^TM^ and the eyelid closed by suture. Mice recovered for an additional 8 weeks to ensure optic nerve degeneration.

### Preparation, Chemical Treatment, and Single Unit Activity Recording of Brain Slices

Brain slices were prepared during the day, ≥2 h before the onset of the dark phase as preparation at night can alter clock phase (Gillette, 1986). The hypothalamus was blocked from the virgin brain and coronal slices were cut at 500 μm with a mechanical chopper. Slices containing the SCN were placed at the interface of a brain slice chamber (Prosser and Gillette, 1991) where they were perfused with glucose-/bicarbonate-/gentamicin-supplemented Earle’s balanced salt solution (EBSS, Gibco BRL/Invitrogen, Carlsbad, CA, United States) while exposed to 95% O_2_:5% CO_2_ at 35°C. This perfusion media was also used as the vehicle of chemical treatment of slices. Spontaneous single unit activity (SUA) was recorded extracellularly, under direct visual guidance of the recording electrode. SCN maintained in this way continue to generate a circadian rhythm of neuronal activity for up to 3 days in explant culture. The SCNs are clearly visible as translucent, ovoid structures at the base of the third ventricle, nestled in the optic chiasm.

Chemical treatment of a slice was applied via microdrop application under visual guidance. Before treatment, media flow was stopped and the media level in the slice chamber was lowered to expose the surface of the SCN. A 1 μl drop was applied to each SCN (two drops/slice) and the cover of the chamber was replaced to prevent evaporation. Glutamate was applied at 10 mM, a dose repeatedly shown to elicit consistent shifts in the mammalian SCN slice (Ding et al., 1994). PACAP was applied at varying concentrations, indicated in context. All treatments were dissolved in fresh, warm, oxygenated perfusion medium (supplemented EBSS) as a vehicle. Following 10 min after stimulus application, the slices were rinsed with fresh perfusion medium, and media flow was restored. At least 1 h was allowed to elapse following treatment before recording was resumed.

The spontaneous extracellular activity of neurons in the SCN was measured by means of a glass electrode filled with a 5-M NaCl solution. The electrode was positioned over the SCN and is advanced slowly through the tissue. When a neuron was detected, its activity was recorded for 4 min. After each recording, the electrode was advanced until another neuron could be found or the slice had been fully penetrated. After a complete pass, the electrode was arbitrarily repositioned so as to sample the entire SCN area. Ensemble activity was determined by means of a 2-h running average grouped into 15-min bins. The peak of the running average was determined visually.

For electrical stimulation (rat), slices were stimulated using a suction electrode or a platinum bipolar electrode. To stimulate the RHT, optic nerves were pulled into the suction electrode or laid across the bipolar electrode. Nerves were stimulated for 5 min, and pulse frequency was set at 5, 10, and 20 Hz. Voltage strength was 2.5–10 V with a 0.05–1 ms pulse duration. SCN population spikes were observed during stimulation to confirm presence of P and N waves associated with post-synaptic responses and to detect possible current spread. If no P and N waves were detected, it was assumed that the nerves were damaged and that slice was not used for recording. Post-recording nerve crush followed by re-stimulation verified elimination of P and N waves. To verify the specificity of RHT activation on the circadian clock, a separate set of experiments stimulated the posterior hypothalamic areas instead of the optic nerves. A bipolar electrode was directed to the posterior hypothalamic area and the tissue was stimulated using the same stimulation parameters as those for the optic nerves.

### Immunoreactivity of PACAP Releasate

Horizontal slices (400–450 μm) containing: (1) the ventral hypothalamus including the SCN, optic chiasm, and optic nerves or (2) a reduced slice (“nerve/chiasm” preparation) containing the SCN, optic chiasm, and optic nerves were prepared between CT 0–2 from Long-Evans rats using a vibrating slicer. Slices were incubated in EBSS supplemented with 24.6 mM glucose, 26.2 mM sodium bicarbonate plus 2.5 mg/l gentamicin, at pH 7.4. A protease inhibitor (Complete^TM^, no EDTA, Roche Diagnostics) was added to the EBSS to prevent proteolysis. Slices stimulated in 0 Ca^2+^ were incubated in calcium-free EBSS, with Complete^TM^ containing EDTA, Roche Diagnostics).

Nitrocellulose filter paper was pre-wetted with 10–20 μl of EBSS to prevent slices adhering to the paper. Slices were placed SCN-side down on the paper and optic nerves were stimulated for 2 min with a bipolar electrode. Slices were gently removed, and the nitrocellulose was immediately fixed in paraformaldehyde vapor for 120–150 min and processed as previously published (Reimer et al., 1999). Briefly, the fixed paper was then incubated with the PACAP antibody (diluted 1:5) for 24 h at 4°C, and then washed in PBS + 0.1% Triton X-100 and incubated with a biotinylated rabbit anti-mouse antiserum (E464, Dako, Copenhagen, Denmark, diluted 1:800) for 1 h. After washing and incubating for 30 min at room temperature in ABC–streptavidin horseradish peroxidase complex diluted 1:125 (Dako, Denmark), the filter papers were washed, then incubated in biotinylated tyramide using a TSA-kit (Tyramide System Amplification; DuPont NEN., Boston, MA, United States) diluted 1:100. After another wash in PBS + 0.1% Triton X-100 and a new incubation in ABC–streptavidin horseradish peroxidase complex as before, the papers were incubated in a solution of diaminobenzidine (DAB, Sigma, St. Louis, MO, United States) for 15 min. The reaction was terminated by washing the filter paper with tap water. Samples on each panel were processed for PACAP together. Images were cropped but not adjusted for brightness or contrast.

Relative staining intensity was quantified using NIH Image J software. Images of the nitrocellulose paper containing the “slice” and the PACAP “stain” were stacked together and an outline of the slice was traced. The stain image intensity was measured, using the slice image outline. Staining intensity was normalized against a 10-μM PACAP-38 microdrop control image. Values are reported as means ± standard error.

### Western Immunoblot

Suprachiasmatic nucleus slices were prepared as previously described, but were then reduced in size to an “SCN punch” by means of a 2-mm diameter sample corer. After treatment, punches were frozen on dry ice and subsequently homogenized by repeat pipetting in 50 μl ice-cold Tissue Protein Extraction Reagent (T-PER) with 1× Complete Protease Inhibitor Cocktail (PI, Roche) and 1× Phosphatase Inhibitor Cocktail (PhI, Cal Biochem, sets I and II). Samples were centrifuged at 10,000 rpm for 5 min at 4°C, and the protein supernatant transferred to a new chilled microfuge tube. Protein content of each sample was determined by the Micro BCA Protein Assay. Total protein (35 μg) was resolved by 4–15% SDS-PAGE and transferred to nitrocellulose. Each blot was probed with rabbit polyclonal antibodies to phosphorylated CREB and rabbit polyclonal anti-CREB (non-phospho-specific) from Upstate Biotechnology, Inc. (Lake Placid, NY, United States). HRP-linked antibodies against rabbit were used in conjunction with Supersignal chemiluminescent substrate (Pierce). Blots were digitally quantitated using the Biochemi Imaging system (UVP, Upland, CA, United States) and Labworks 4.0. pCREB-ir was compared to total CREB-ir to control for loading differences between wells, and this ratio was used to determine CREB phosphorylation. Average CREB phosphorylation in untreated slices of each blot was normalized to 1 to facilitate comparisons of induction.

### Double Immunofluorescence

Immunofluorescence staining of coronal sections through the anterior diencephalon of FFPE brains of C57Bl/6 mice was carried out as previously described (Schafer et al., 2010), using the antibodies described and at the dilutions previously employed. Immunofluorescence signals were documented in a surface scan using a BX50WI confocal laser scanning microscope (Olympus Optical, Hamburg, Germany) and Olympus Fuoview 2.1 software, and stored as false color images (8-bit tiff format).

### RNAScope Analysis of PACAP mRNA Expression in Retina

The eyeballs from three wild-type mice (C57BL/6N, *n* = 3) were freshly excised at each time point, frozen on Dry Ice, embedded in freezing medium (TissueTec^TM^) at −20°C and sectioned on a cryostat. Sagitta examine PACAP mRNA expression in retina by *in situ* histochemistry. The RNAscope 2.5 HD Reagent Kit-RED assay (Cat No. 322360, Advanced Cell Diagnostics, San Francisco, CA, United States) and mouse PACAP probe (Adcyap1, Cat No. 405911, Advanced Cell Diagnostics, San Francisco, CA, United States) were used for *in situ* hybridization of mouse retina sections following the user manual of the products. 50% hematoxylin staining solution was used for counterstaining. Slides with retina sections were image-captured with 20× objective with ZEISS Axio Scan (Carl Zeiss Microscopy, Thornwood, NY, United States) and images for sections from each animal were organized and converted to TIF files with BrainMaker (MBF Bioscience, Williston, VT, United States).

### Data Quantitation and Statistical Analysis

Data for immunohistochemical investigations are presented as representative images to justify qualitative and comparative statements made in text about chemoanatomical features of PACAP circuitry. Single-plex images of RNAscope results were quantified with Fuji ImageJ. Briefly, color deconvolution was used to separate the single-plex images into different color channels: images for counter-stained nuclei were used to count cell number on retinal ganglion cell (RGC) layer and images for PACAP probe signals (RED) were used to quantify the integrated density (IntDen) of the signal within the RGC layer. Relative intensity of PACAP mRNA signal/cell at each time point was calculated as: IntDen of probe signal divided by cell number, then normalized by mean of the IntDen/cell at 11 AM (CT5). *Post hoc* Bonferroni analysis following one-way ANOVA using SigmaPlot 14.0 (Systat Software, Inc., San Jose, CA, United States) was conducted to compare PACAP mRNA expression between different time points.

For electrical recordings, the average time of peak activity of the neuronal ensemble was compared between each treatment and untreated animals to test the presence of a phase shift, and between experimental (PACAP-null, enucleated), and control (wild-type) animals for each condition using ANOVA. For western immunoblot, normalized densitometry was compared between control and each condition using ANOVA. Statistics are reported as the mean ± SEM. Alpha was set at 0.05 for all statistical tests.

## Results

### PACAP Release From the Retinohypothalamic Tract Onto the SCN and Phase Advance Elicited by Optic Nerve Stimulation Exhibit Identical Frequency-Dependence

**FIGURE 1:**
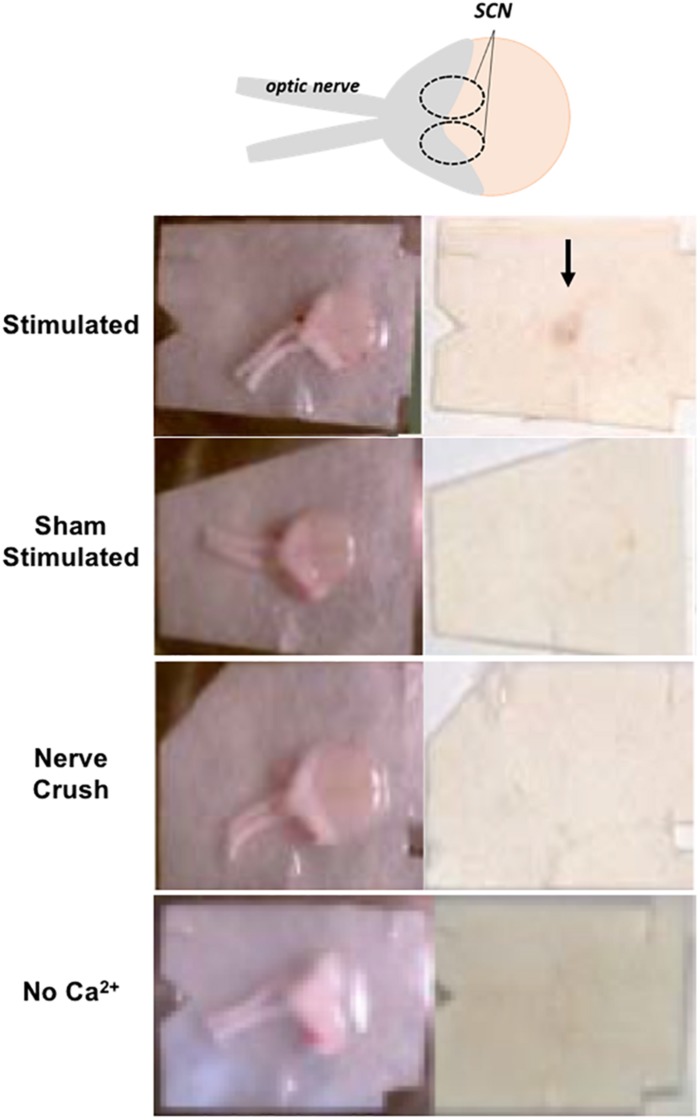
PACAP release after optic nerve stimulation is calcium- and frequency-dependent. PACAP releasate is immunodetected in the SCN region of horizontal brain slices following optic nerve stimulation but not in sham stimulated, simulation following nerve crush, or in bathing medium lacking external Ca^2+^.

A 20 Hz, 5 V, 2 min optic nerve stimulation invokes a phase advance in rodent horizontal brain slices (Atkins et al., 2018). Accordingly, to determine a correlation indicative of causality between PACAP release and phase advance elicited in the SCN from the retinohypothalamic tract (RHT), we performed PACAP release experiments in rat SCN *ex vivo*, a preparation in which peptide secretion could technically be readily monitored. PACAP staining specific to the SCN was examined by using reduced horizontal slices containing the optic nerves, the optic chiasm, and its overlying hypothalamic tissue, including the SCN. PACAP release experiments revealed that 20 Hz stimulation to the optic nerves released PACAP (Figure 1). To determine if RHT-activated PACAP release was synaptic in nature, samples were also stimulated in the absence of external calcium, or after optic nerve crush. PACAP staining intensity was not different among sham-stimulated, 20 Hz in 0 Ca^2+^, and nerve crush followed by 20 Hz stimulation samples. Only 20 Hz stimulated samples with normal extracellular calcium and intact optic nerve input exhibited significantly higher levels of PACAP staining compared to sham-stimulated controls (Figure 1).

The results above establish a correlation between firing frequency of the retinohypothalamic inputs to the SCN, and PACAP release within the SCN itself. Thus, the circadian dependence of glutamate administration to trigger late-night phase shifting would be expected to show PACAP dependence in a system in which endogenous PACAP expression could be independently manipulated. We turned to the mouse *ex vivo* SCN slice preparation in order to answer this question.

### Distribution and Glutamatergic Phenotype of PACAPergic Fibers to SCN in the Mouse

**FIGURE 2:**
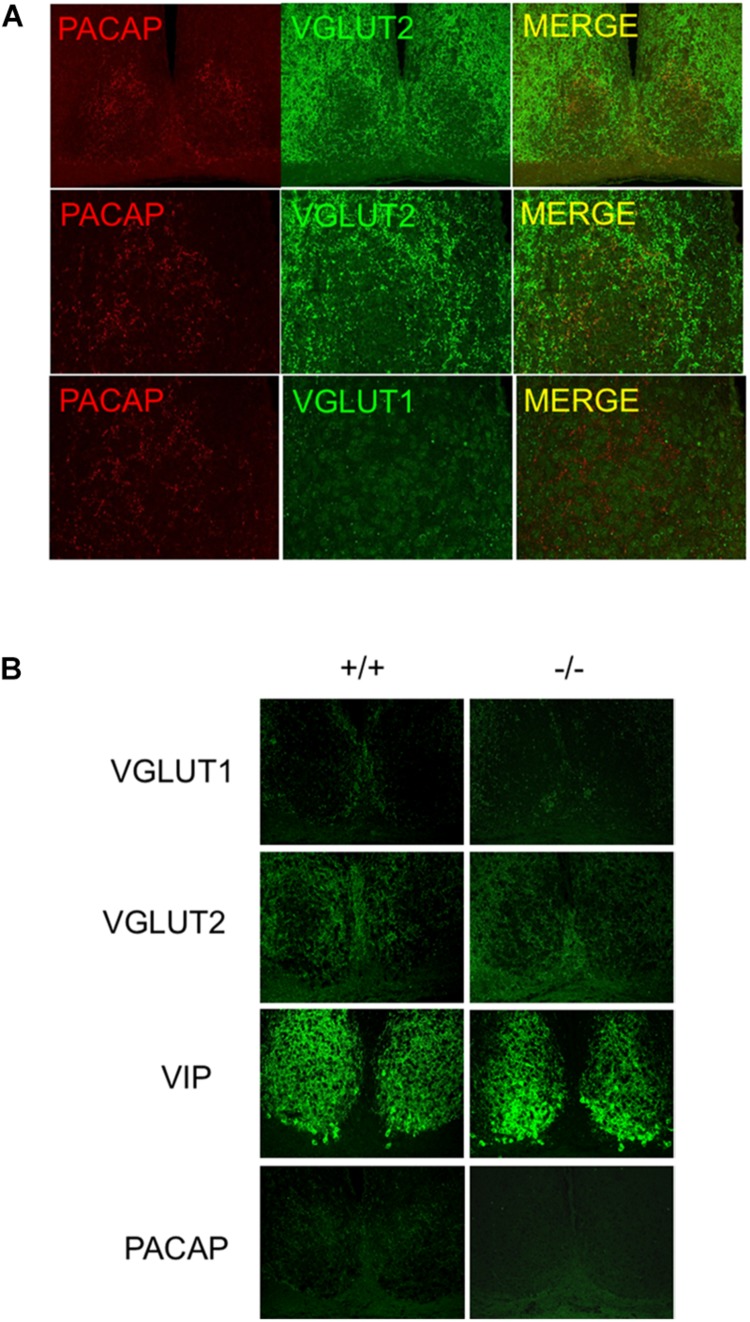
Distribution of VGluTs, PACAP, and other neurotransmitters in mouse SCN. **(A)** PACAP co-localization with VGluT2 in afferent nerve terminals to SCN. Micrographs show PACAP co-localized with VGluT2 not VGluT1 in SCN. **(B)** PACAP terminal loss in SCN in PACAP-deficient mice. the panels show antibody specificity and lack of perturbation of VIP or VGluT staining in SCN in absence of PACAP.

Pituitary adenylate cyclase-activating polypeptide innervation of the mouse SCN is coincident with the expression of VGLUT2 in SCN terminals (Figure 2A). Importantly, after removal of PACAP (i.e., in PACAP-deficient mice) the pattern of innervation by VGluT2, and the pattern of expression of VIP in the cells of the SCN itself is unchanged (Figure 2B), demonstrating the SCN in PACAP-deficient mice remains essentially unchanged, save for the absence of PACAP itself. In addition, RHT innervation of the mouse SCN appears similar to that of other rodent species, and justifies at least limited generalization about the pattern and purpose of retinohypothalamic innervation of the SCN originating in intrinsically photosensitive retinal ganglion cells (ipRGCs) (Keenan et al., 2016).

### Spontaneous Electrical Firing Rate Shifts Mirror Behavioral Rhythm Activity of SCN in the Mouse

**FIGURE 3:**
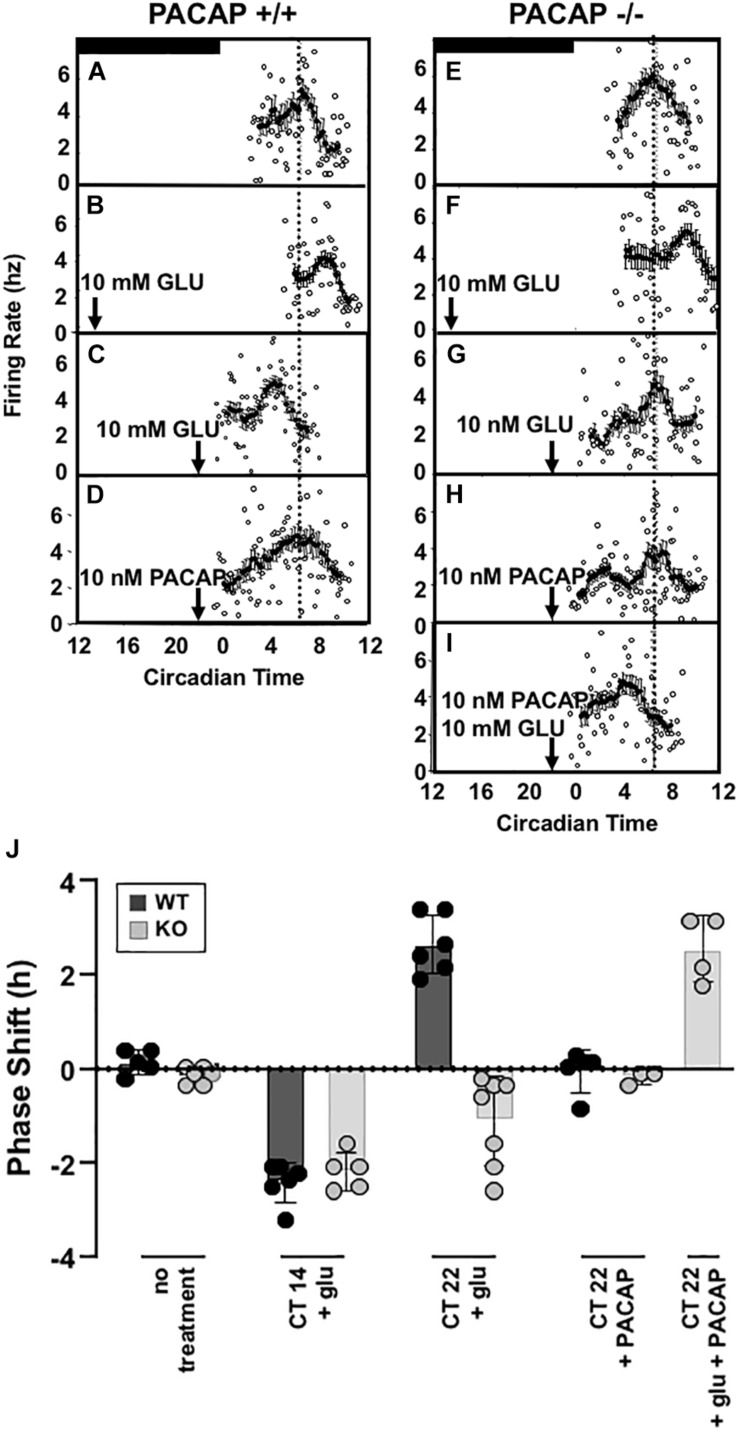
Glutamate-stimulated phase advance in SCN slices is absent in PACAP-deficient mouse slices and is rescued with exogenous PACAP. Left panel **(A–D)** shows the phase advancing and delaying effects of late and early night glutamate application in a representative *ex vivo* hypothalamic/SCN slice preparation, and the lack of effect of PACAP alone on circadian rhythm of electrical activity of the SCN. Right panel **(E–I)** shows the lack of phase advance by late-night glutamate application in a representative *ex vivo* hypothalamic/SCN slice preparation from PACAP-deficient mice, and its rescue by application of exogenous PACAP, which applied alone is without effect on phasing of neuronal firing. **(J)** (separate panel) this summarizes the phase shifts of all preparations tested (i.e., from four to seven individual animals/*ex vivo* preparations per condition), calculated as described in the section “Materials and Methods.”

Firing-rate patterns can be used to determine subjective time following chemical treatment of SCN that have been removed from mice and maintained in culture. This allows the examination of phase shifting following chemical application directly to SCN, despite the absence of wheel – running activity. As in behavioral experiments, untreated slices of wild-type, biorbitally enucleated wild-type, and PACAP-deficient mice were recorded to determine whether and when each showed a spontaneous peak of firing-rate frequency. Each exhibited peaks between CT 6 and 7 [wild-type: CT 6.25 ± 0.12 h, PACAP-null: CT 6.54 ± 0.07 h (Figure 3); enucleated: CT 6.58 ± 0.17; mean ± SEM, *n* = 5–6]. These are consistent with previous electrical recordings in rat and mouse *ex vivo* hypothalamic slices (Green and Gillette, 1982; Shibata et al., 1982; Prosser et al., 1989; Burgoon et al., 2004). With these baseline characteristics established in the brain-slice preparation, we wished to determine whether exposure to glutamate would induce shifts in firing-rate patterns in slices, as exposure to light shifts wheel-running patterns in intact animals.

Applying glutamate to SCN of wild-type and PACAP-null mice is an approximation of natural phase shifting, but one step further “downstream” from simple light exposure. By isolating and exposing the SCN, neurotransmitters such as glutamate may be applied and SCN activity observed directly. To test whether or not PACAP-null mice would show intact shifts to direct glutamate application, we examined the glutamate responsiveness of wild-type and PACAP null mice at selected time-points of the early (CT 14) and late (CT 22) night, to determine if the PACAP dependence of light-induced phase advance in circadian rhythm we reported in intact mice would persist in the *ex vivo* preparation. In that case, potential indirect effects of PACAP deficiency on SCN function, such as impaired sensory transduction from the retina secondary to pupillary reflex impairment, could be eliminated. SCN from wild-type mice respond to glutamate during the subjective night in a manner parallel to the response of intact animals to light in the mouse, as in the rat (Ding et al., 1994), with significant phase delays at CT 14 (Ding et al., 1994) and phase advances at CT 22 (2.81 ± 0.26 h, *n* = 5, Figure 3).

Consistent with results from wheel-running experiments, PACAP-deficient mice showed robust phase delays (131.5 ± 11.2 min, *n* = 5) in response to glutamate stimulation at CT 14. These delays were significantly different from untreated slices (*p* < 0.001 for each, *t*-test) but not significantly different from shifts seen in SCN, from wild-type mice, treated with glutamate at CT14 (*p* > 0.2, n.s., *t*-test). In contrast to the shifts seen in early night, and consistent with behavioral results, SCN from PACAP-null mice failed to show the robust phase advance response to application of glutamate at CT22 seen in slices from wild-type mice. Thus, PACAP-deficient mouse slices showed phase delays in response to glutamate at CT14 (75.5 ± 23.8 min, *p* < 0.001), but not phase advances in response to glutamate at CT22 (Figure 3). These shifts replicate alterations in light-responsiveness in intact, behaving mice, supporting the use of the brain-slice preparation to study further the mechanisms of PACAP-glutamate co-transmission in phase modulation of SCN rhythmicity.

### Exogenous PACAP Rescues Late Night Phase Advancing Activity in PACAP-Null Mice

The results described above strongly suggest that PACAP plays an integral role in phase advances in response to light and glutamate during late night phase advance, but not early night phase delay. This is largely consistent with results seen in PAC1-R null mice (Hannibal et al., 2001a), but is not the same as results seen in other PACAP-null mouse models which demonstrate a diminished phase delay in early night (Kawaguchi et al., 2003; Colwell et al., 2004). One of the potential confounds of any genetically ablated animal model is the potential for wide-ranging, non-specific alterations as a result of a genetic lesion present during the entire course of development. An alternate explanation for altered shifting might suggest that such non-specific developmental disruptions result in altered shifting in the absence of a critical role for PACAP during the normal course of phase resetting. Fortunately, the PACAP-null mouse model holds the capacity to test such a hypothesis. Because exogenous PACAP may be replaced in the brain-slice preparation, the potential for PACAP to restore normal responses to glutamate may be directly tested.

A 1 μl drop of 10 nM PACAP does not shift either wild-type or PACAP-null SCN slices when applied alone at CT 22. Peak times were not distinguishable from untreated slices (*p* > 0.2, not significant, Figure 3E). It has already been seen that 10 mM glutamate induces phase delays in PACAP-null mice. However, 10 nM PACAP, applied with 10 mM glutamate at CT 22, restored phase advances in PACAP-null mice that were significantly different than both untreated slices and slices treated only with PACAP, but not statistically different than those seen in wild-type mice (shift = 153.9 ± 21.1 min, *p* > 0.2, Figure 3).

Data accrued for individual experiments shown in Figure 3 are summarized in Figure 3J, where the overall early and late-night phase shifts induced by glutamate in wild-type and PACAP-null mice, and the effects of PACAP in rescuing defects in each case, are depicted graphically.

### Dose–Response of PACAP Replacement on Glutamate-Induced Phase Shifting in PACAP-Null Mice

**FIGURE 4:**
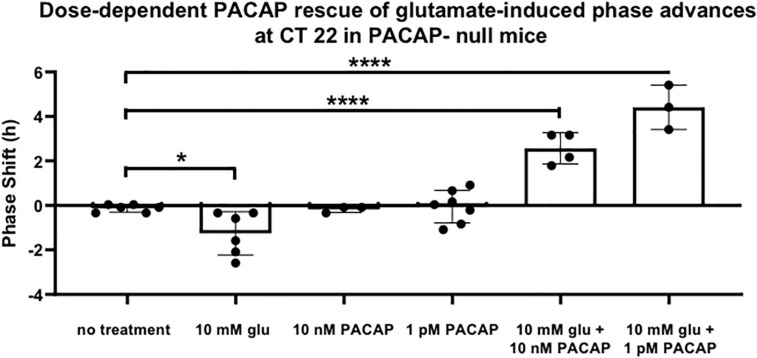
PACAP rescues phase advances in PACAP-null mice with magnitudes that are dose-dependent. Neither glutamate nor PACAP induce phase advances when applied alone to PACAP-null SCN brain slices at CT 22. When they are applied together, they induce phase advances that are inversely related to PACAP dose. One-way ANOVA, Dunnett’s *post hoc* test. ^∗^*p* < 0.05, ^****^p < 0.0001.

In order to examine glutamate signaling in the absence of confounding effects of PACAP, glutamate and PACAP were co-applied in PACAP-null mice using 1 pM and 10 nM PACAP concentrations, doses that had previously been shown to have no significant shifting effect on the clock. 10 nM PACAP, applied with 10 mM glutamate at CT 22, restored phase advances in PACAP-null mice that were not statistically significantly different than those seen in wild-type mice (shift = 153.9 ± 21.1 min, *p* > 0.2). Co-application of glutamate and 1 pM PACAP also resulted in phase shifts, but these shifts were substantially larger than those induced by glutamate in wild-type mice (shift = 264.5 ± 34.6 min). These shifts were significantly larger than those seen either in glutamate-treated wild-type mice (*p* ≤ 0.014) or PACAP-null mice exposed to glutamate and 10 nM PACAP (*p* ≤ 0.035) (Figure 4).

### Adult Lesion of PACAP via Biorbital Enucleation Demonstrates Similar Results to the PACAP-Null Mice

**FIGURE 5:**
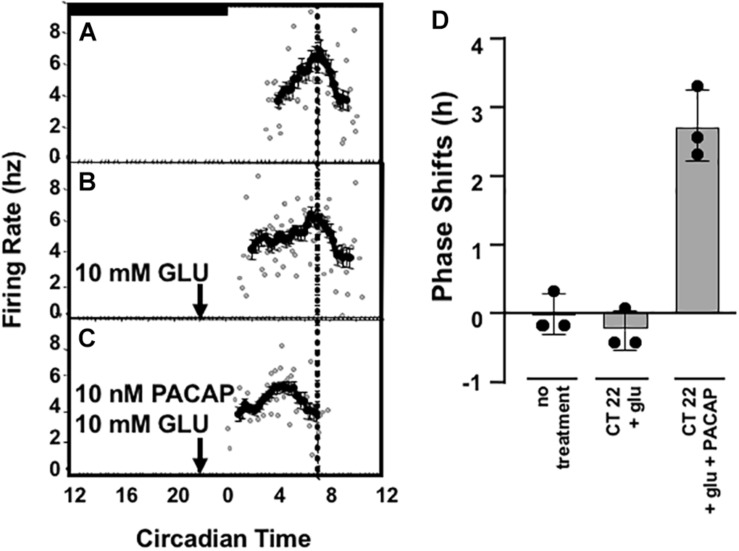
Summary of PACAP dependence of late-night phase advance in slices from enucleated wild-type C57Bl/6 mice. Panels **(A**–**C)** depict PACAP dependence of glutamate-induced late-night phase advance using the doses of glutamate and PACAP shown to best mimick physiological phase-advance (i.e., phase advance observed *in vivo* in C57Bl6 mice). Panel **(D)** summarizes the phase shift data obtained from preparations from three separate animals for each treatment condition. In enucleated mice, treatment with glutamate + PACAP is significantly induced as compared to no treatment or with glutamate alone. *p* < 0.001, One-way ANOVA, Tukey *post-hoc* test.

The importance of the retinal input of PACAP, rather than PACAP innervation from other sources, on the phase advance is demonstrated by the lack of a phase advance with glutamate application at CT 22 in SCN from biorbitally enucleated mice. These displayed no significant difference in their peak in firing rate between untreated (CT 6.58 ± 0.17) and those treated with a 10 mM glutamate application at CT 22 (CT 6.83 ± 0.20), unless PACAP was added to the preparation during glutamate administration (CT 3.83 ± 0.30) (Figure 5).

### Glutamate-Induced pCREB Induction Is Attenuated in PACAP-Null Mice

**FIGURE 6:**
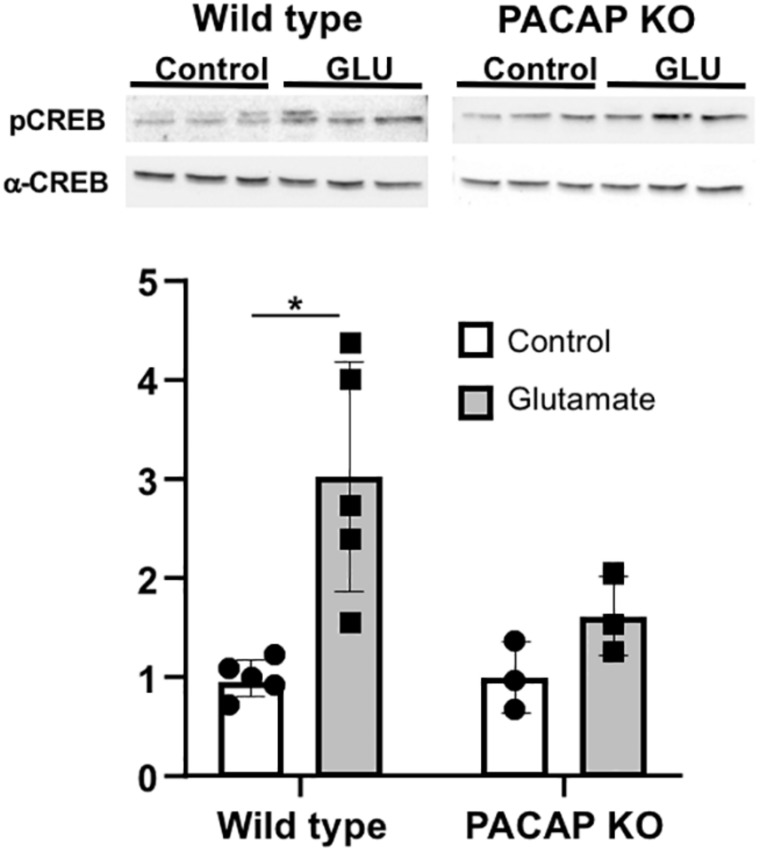
Glutamate-induced stimulation in CREB phosphorylation is attenuated in PACAP-null animals. PCREB immunoreactivity normalized to total CREB was significantly induced in SCN with glutamate in wild-type animals. Induction in PACAP-null mice is lower than in wild-type mice. Number of slices per group indicated on each bar (wt = 5; ko = 3) Two-way ANOVA; Tukey *post-hoc* test. ^∗^*p* < 0.05.

As seen in other rodents (Ginty et al., 1993; Ding et al., 1997; McNulty et al., 1998; von Gall et al., 1998; Tischkau et al., 2003), glutamate treatment in wild-type mice elicited a significant two to threefold induction in phosphorylation of CREB (2.69 ± 0.51-fold induction vs. untreated *p* ≤ 0.01, *n* = 5). In PACAP-null mice, a slight induction was noted, although this induction was not significant. The lack of induction of pCREB in PACAP-null mice demonstrates a role for PACAP in the known internal signal transduction pathways stimulated by glutamate (Figure 6).

### PACAP mRNA Expression in Retina Across the Circadian Period

**FIGURE 7:**
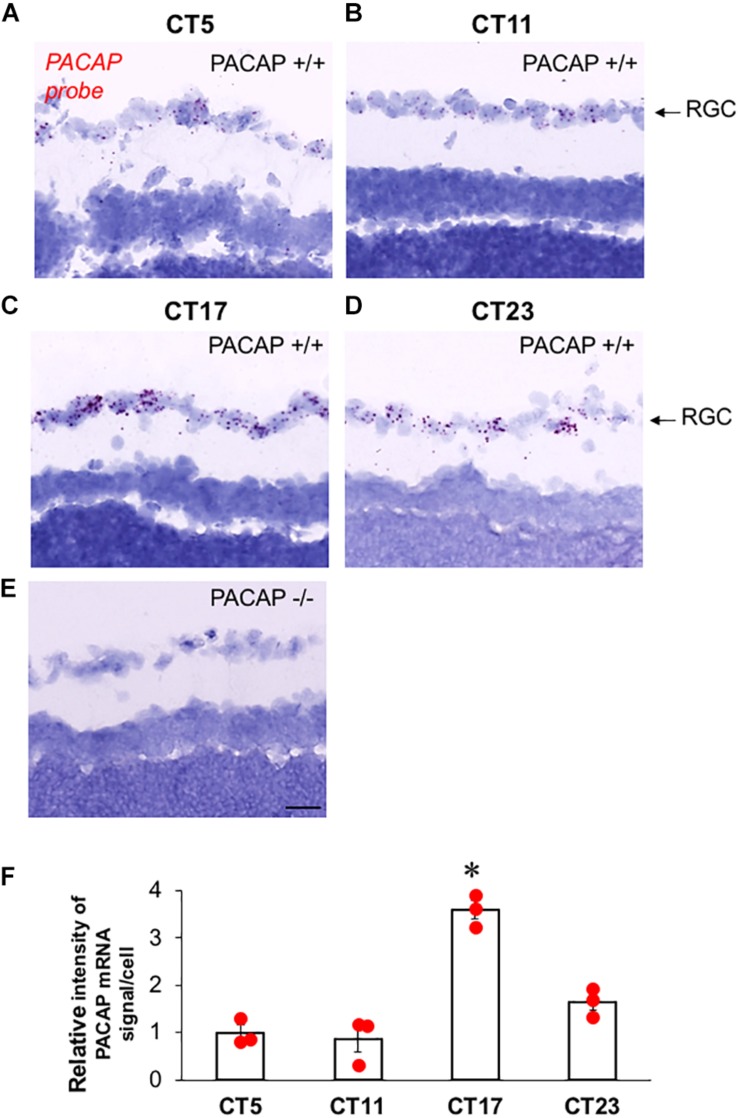
PACAP mRNA expression in ipRGCs of C57Bl6 mice during late subjective night, and mid-subjective day. **(A–D)** Representative photomicrograph of sagittal section from wild-type (PACAP+/+) C57Bl6 mouse retina at CT5–CT23 with PACAP transcripts (red) in retinal ganglion cell (RGC) layer. **(E)** Representative photomicrograph of sagittal section from PACAP KO (PACAP–/–) mouse retina at CT23 with invisible PACAP transcripts in RGC layer. **(F)** Relative expression level of PACAP mRNA in RGC at CT5, CT11, CT17, and CT23. The scatter plot was from three animals per time point. The box and error bar represent mean ± SEM. One-way ANOVA followed by *post hoc* Bonferroni analysis: ^∗^*p* < 0.01, CT17 compared to CT5, CT11, or CT23. Scale bar: 25 μm.

The expression of endogenous PACAP in the retina has not previously been examined in C57Bl6 mice, although previous studies in PACAP-deficient and wild-type C57Bl6 mice have assumed that PACAP mRNA and peptide are expressed in ipRGCs of mice, and in this mouse strain, as previously reported in the rat, and as well, EGFP expression is found in ipRGCs of PACAP-EGFP transgenic mice (Condro et al., 2016). Surprisingly, we found a wide range in the intensity and number of PACAP mRNA-positive cells in the retina sampled at various times, and performed a systematic study across the circadian period to characterize this variation. The number of PACAP mRNA-positive ipRGCs increases dramatically during subjective night, compared to subjective day in the C57Bl/6N strain used in these studies (Figure 7). Changes in PACAP expression across the day have not previously been reported in any rodent species including mouse and rat, and further examination of this phenomenon in both rats and mice appears warranted.

## Discussion

The ability of the clock to entrain to external light and darkness is dependent upon photic transmission from eyes to the SCN via a subset of RGCs. Glutamate is accepted as a first message carrying light information from the RGCs to the SCN; however, the distribution of melanopsin in the RGCs reaching the SCN was also found to be identical to that for PACAP (Hannibal et al., 2002), implying a role for PACAP in light-entrainment of the clock. Investigations of PACAP and glutamate in circadian photic entrainment have revealed that these neuromodulators are co-stored in RGCs with innervation in the SCN (Hannibal et al., 2000).

Here, we have extended the detailed examination of the PACAP dependence of light-induced phase shifting *in vivo*, to the SCN slice preparation *ex vivo*. Firing-rate patterns are used to determine subjective time following chemical treatment of SCN that have been removed and maintained *ex vivo*, allowing examination of phase shifting following chemical application directly to SCN tissue. Applying glutamate to SCN slices, *ex vivo*, of wild-type and PACAP-null mice is an approximation of natural phase shifting one step removed from retinal light exposure. Overall, phase shifts in electrical activity replicate alterations in light-responsiveness in intact, behaving mice.

We show here that in the mouse SCN *ex vivo*, glutamate drives both late-night and early-night light exposure effects on circadian rhythm, and that the effect on late-night phase advance, as in the mouse *in vivo*, is PACAP-dependent, demonstrating that these phase-shifting modulatory effects of PACAP are dependent not on visual, but on non-visual photic sensory input to the SCN. The major expression of PACAP in the SCN is due to efferent connections from the retina via the RHT (Hannibal et al., 1998; Hannibal and Fahrenkrug, 2004). Severing the optic nerves and allowing degeneration of those nerves produces a PACAP “knockout” limited to the retinohypothalamic input to the SCN *in vivo*, and subsequently in the SCN-containing hypothalamic slice. We have recapitulated the effects of PACAP deficiency in hypothalamic slices from enucleated wild-type mice, in which retinohypothalamic inputs have degenerated, to demonstrate that it is specifically PACAP supplied from these inputs, rather than from other hypothalamic regions projecting to SCN, that is responsible for the modulation of glutamate-dependent non-visual photic regulation of circadian rhythmicity in SCN.

The results obtained here strongly suggest that PACAP plays an integral role in wild-type phase advances in response to light and glutamate during the late night. Our findings are inconsistent with a critical role of PACAP during the early night, as PACAP-null mice respond to light and glutamate with the same direction and magnitude of shift as wild-type mice despite the lack of PACAP expression. This is largely consistent with results seen in PAC1-R-deficient mice (Hannibal et al., 2001a), but is not the same as results seen in other PACAP-deficient mouse models (Kawaguchi et al., 2003; Colwell et al., 2004). One of the potential confounds of any genetically ablated animal model is the potential for wide-ranging, non-specific alterations as a result of a genetic lesion present during the entire course of development. An alternate explanation for altered shifting might suggest that such non-specific developmental disruptions result in altered shifting in the absence of a critical role for PACAP during the normal course of phase resetting. Fortunately, the PACAP-deficient mouse model holds the capacity to test such a hypothesis. Because exogenous PACAP may be replaced in the brain-slice preparation, the potential for PACAP to restore normal responses to glutamate could be directly tested.

The data from these experiments are furthermore consistent with a surprisingly specific sensitivity of the SCN to PACAP. The circadian clock, although able to maintain a near-24-h rhythm and appropriately delay during the early night, is nonetheless critically damaged during the late night in mice lacking PACAP. Replacing PACAP directly on the SCN brain slice, at only the time of glutamate application, and only in the area of the SCN, is sufficient to rescue wild-type shifting in the presence of glutamate. The most parsimonious conclusion supported by these data is that PACAP (or part of its signaling cascade) interacts with glutamate to shift the endogenous rhythm of the mammalian SCN during the late night. It will be of particular interest to examine in the *ex vivo* hypothalamic explant preparation the phenocopying of phase-shifting effects of downstream components of glutamate signaling, such as cAMP and cGMP signaling (Tischkau et al., 2000, 2003), and their PACAP dependence, as this should provide valuable insights into how PACAP acts post-synaptically to allow glutamate phase-advance to occur in this preparation and, presumably, *in vivo*.

It will also be of critical interest to compare the effects of PACAP on pupillary constriction in the eye, apparently independently of glutamate transmission, and phase-shifting at the level of the SCN. Is PACAP released all night, or only when the SCN seems to require it? This question is of particular relevance in view of our novel observation of a circadian rhythm to the expression of PACAP itself (i.e., its mRNA) in the ipRGC, with significantly higher levels during subjective night than during subjective day. What is the intracellular response of PACAP as a function of time of day, as its receptor(s) appear to be present throughout the day, and how does it interact with that of glutamate? Do all (or any) light pulses cause PACAP release, and when throughout the day is PACAP actually available for release in response to photic stimulation? The experimental system employed here affords the opportunity to study the nature of co-transmission in advances and delays more intimately, as well as to obtain greater insight into the mechanisms of species differences in phase shifting in response to light.

It is worthy of comment that the altered phase-shifting seen in these experiments reported here is similar to the results originally reported regarding mice with an altered PACAP receptor (PAC1-R). PAC1-R-null mice showed early-night responses that were similar to wild-type mice (although light-pulses at CT 20 induced longer delays in the PAC-1-R null mice). During the late night (CT23), however, PAC1-R-nulls showed phase delays opposite to the advances seen in wild-type mice (Hannibal et al., 2001a). These data are consistent with our previous results (Beaule et al., 2009).

How does PACAP modulation of glutamate signaling in SCN, and therefore potentially at other CNS synapses, compare to what is known about PACAP signaling from cell culture and other experiments? We have reported preliminarily (Zhang et al., 2018; Society for Neuroscience Annual Meeting, San Diego, CA, United States, November 2018) that PACAP seems to act downstream of glutamate signaling for late-night phase advance, in that the phase-shifting effects of cGMP, elevated by and mimicking the *ex vivo* effects of glutamate itself, are likewise blocked in PACAP-deficient slices, and this impairment is removed by direct application of PACAP together with glutamate at subjective late-night in the mouse SCN *ex vivo*. Other reports suggest that PACAP acts (Darvish and Russell, 1998; Chen et al., 1999; Michel et al., 2006) via non-specific calcium channel opening, downstream of elevation of cAMP, acknowledged to be the major intracellular effect of stimulation of the PAC1, VPAC1, and VPAC2 receptors through which PACAP acts. The fact that the modulatory effects of PACAP in “wild-type” SCN slices *ex vivo* can be restored, in PACAP-deficient mice, by addition of exogenous PACAP strongly suggests that, at least in the SCN, the effects of PACAP may be mediated either by glutamate-stimulated PACAP release at intact retinohypothalamic (psRGC) nerve terminals in the *ex vivo* preparation, or that a low level of spontaneous PACAP release is sufficient to “prime” late-night phase advancing effects within the SCN. In addition to concerted post-synaptic effects of glutamate and PACAP at this and other synapses, the possibility exists that PACAP modulates glutamate effects post-synaptically via PAC1-mediated activation of astroglial inputs at the post-synapse (Miyata Review, ANYAS, in press). PACAP also acts directly, in neurons and endocrine cells, to activate the mitogen-activated protein kinase ERK (Frödin et al., 1994; Barrie et al., 1997; Hashimoto et al., 2003; Butcher et al., 2005; Gerdin and Eiden, 2007; Ravni et al., 2008; Emery and Eiden, 2012; May et al., 2014), and this has recently been shown to occur via a novel cAMP effector, NCS-Rapgef2 (Emery et al., 2013; Missig et al., 2014, 2015). In any event, it seems that PACAP signaling may well shape glutamate responsivity, in addition to its own potential wholly independent effects at synapses at which there is no evidence for glutamate co-transmission. It remains for future research to link glutamate/PACAP co-signaling to the effects of PACAP on metaplasticity in hippocampus (Macdonald et al., 2005, MacDonald et al., 2007; Costa et al., 2008), in fear processing in amygdala and hippocampus (Hammack and May, 2015; Schmidt et al., 2015), cocaine relapse in BNST (Miles et al., 2016, 2017), and multiple effects in projections from frontal cortex (Crestani et al., 2013).

As mentioned above, identifying the possible locus of PACAP action in modulation of glutamatergic signaling in the SCN by examining the PACAP dependence of late-night phase-shifting by downstream messengers activated by glutamate in SCN will be an important next step forward in full understanding of this glutamate-PACAP co-modulatory system (Gillette and Mitchell, 2002). At this time, we suggest that PACAP’s physiological role, regardless of its mode of action, is to augment this signaling pathway during times when glutamate stimulation alone is insufficient. How and why PACAP expression waxes and wanes throughout the day in pigmented retina, and if this pattern is functionally related to PACAP circadian function, is a compelling question for future investigation, both for understanding of the full effects of retinal photosensitivity on sensory information processing and behavior in mammalian species, and for determining if the retinohypothalamic projection to the SCN is paradigmatic for PACAP neurotransmission in other brain regions.

## Data Availability Statement

All datasets generated for this study are included in the article/supplementary material.

## Ethics Statement

The animal study was reviewed and approved by the University of Illinois at Urbana-Champaign and the National Institutes of Health.

## Author Contributions

PL, JM, PB, CB, EW, MS, LE, and SJ contributed to the experimental data. LE, MG, JM, PL, and SJ wrote the manuscript.

## Disclaimer

At least a portion of this work is authored by Lee E. Eiden on behalf of the U.S. Government and, as regards Dr. Eiden and the U.S. Government, is not subject to copyright protection in the United States. Foreign and other copyrights may apply.

## Conflict of Interest

The authors declare that the research was conducted in the absence of any commercial or financial relationships that could be construed as a potential conflict of interest.

## Abbreviations

PACAP: pituitary adenylate cyclase-activating polypeptide
SCN: suprachiasmatic nucleus
VGluT2: vesicular glutamate transporter isoform 2.

## References

[B1] Abbott S. M. (2005). *Regulation of Circadian Rhythms by Sleep-Wake Centers in the Brainstem and Basal Forebrain.* Champaign, IL: University of Illinois.

[B2] Atkins N Jr Ren S. Hatcher N. Burgoon P. W. Mitchell J. W. Sweedler J. V. (2018). Functional peptidomics: stimulus- and time-of-day-specific peptide release in the mammalian circadian clock. *ACS Chem. Neurosci.* 9 2001–2008. 10.1021/acschemneuro.8b00089 29901982 PMC6125129

[B3] Aton S. J. Colwell C. S. Harmar A. J. Waschek J. Herzog E. D. (2005). Vasoactive intestinal polypeptide mediates circadian rhythmicity and synchrony in mammalian clock neurons. *Nat. Neurosci.* 8 476–483. 10.1038/nn1419 15750589 PMC1628303

[B4] Barrie A. P. Clohessy A. M. Buensuceso C. S. Rogers M. V. Allen J. M. (1997). Pituitary adenylyl cyclase-activating peptide stimulates extracellular signal-regulated kinase 1 or 2 (ERK1/2) activity in a ras-independent, mitogen-activated protein kinase/ERK kinase 1 or 2-dependent manner in PC12 cells. *J. Biol. Chem.* 272 19666–19671. 10.1074/jbc.272.32.19666 9242621

[B5] Beaule C. Mitchell J. W. Lindberg P. T. Damadzic R. Eiden L. E. Gillette M. U. (2009). Temporally restricted role of retinal PACAP: integration of the phase-advancing light signal to the SCN. *J. Biol. Rhythms* 24 126–134. 10.1177/0748730409332037 19382381 PMC2914551

[B6] Buchanan G. F. (2002). *Cholinergic Regulation of the Mammalian Circadian System: Analysis of Cholinergic-Induced Phase Shifting in vivo and in vitro in Wildtype and M1 Knockout Mice.* Champaign, IL: University of Illinois at Urbana-Champaign.

[B7] Buchanan G. F. Gillette M. U. (2005). New light on an old paradox: site-dependent effects of carbachol on circadian rhythms. *Exp. Neurol.* 193 489–496. 10.1016/j.expneurol.2005.01.008 15869951

[B8] Burgoon P. W. Lindberg P. T. Gillette M. U. (2004). Different patterns of circadian oscillation in the suprachiasmatic nucleus of hamster, mouse, and rat. *J. Comp. Physiol. A Neuroethol. Sens. Neural. Behav. Physiol.* 190 167–171. 10.1007/s00359-003-0486-z 14714137

[B9] Butcher G. Q. Lee B. Cheng H.-Y. M. Obrietan K. (2005). Light stimulates MSK1 activation in the suprachiasmatic nucleus via a PACAP-ERK/MAP kinase-dependent mechanism. *J. Neurosci.* 25 5305–5313. 10.1523/jneurosci.4361-04.2005 15930378 PMC6724997

[B10] Chen D. Buchanan G. F. Ding J. M. Hannibal J. Gillette M. U. (1999). Pituitary adenylyl cyclase-activating peptide: a pivotal modulator of glutamatergic regulation of the suprachiasmatic circadian clock. *Proc. Natl. Acad. Sci. U.S.A.* 96 13468–13473. 10.1073/pnas.96.23.13468 10557344 PMC23971

[B11] Colwell C. S. Michel S. Itri J. Rodriguez W. Tam J. Lelievre V. (2004). Selective deficits in the circadian light response in mice lacking PACAP. *Am. J. Physiol. Regul. Integr. Comp. Physiol.* 287 R1194–R1201. 15217792 10.1152/ajpregu.00268.2004

[B12] Condro M. C. Matynia A. Foster N. N. Ago Y. Rajbhandari A. K. Jayaram B. (2016). High-resolution characterization of a PACAP-EGFP transgenic mouse model for mapping PACAP-expressing neurons. *J. Comp. Neurol.* 524 3827–3848. 10.1002/cne.24035 27197019 PMC5063673

[B13] Costa L. Santangelo F. Li Volsi G. Ciranna L. (2008). Modulation of AMPA receptor-mediated ion current by pituitary adenylate cyclase-activating polypeptide (PACAP) in CA1 pyramidal neurons from rat hippocampus. *Hippocampus* 19 99–109. 10.1002/hipo.20488 18727050

[B14] Crestani C. C. Alves F. H. Gomes F. V. Resstel L. B. Correa F. M. Herman J. P. (2013). Mechanisms in the bed nucleus of the stria terminalis involved in control of autonomic and neuroendocrine functions: a review. *Curr. Neuropharmacol.* 11 141–159. 10.2174/1570159X11311020002 23997750 PMC3637669

[B15] Cutler D. J. Haraura M. Reed H. E. Shen S. Sheward W. J. Morrison C. F. (2003). The mouse VPAC2 receptor confers suprachiasmatic nuclei cellular rhythmicity and responsiveness to vasoactive intestinal polypeptide in vitro. *Eur. J. Neurosci.* 17 197–204. 10.1046/j.1460-9568.2003.02425.x 12542655

[B16] Darvish N. Russell J. T. (1998). Neurotransmitter-induced novel modulation of a nonselective cation channel by a cAMP-dependent mechanisms in rat pineal cells. *J. Neurophysiol.* 79 2546–2556. 10.1152/jn.1998.79.5.2546 9582227

[B17] Ding J. M. Chen D. Weber E. T. Faiman L. E. Rea M. A. Gillette M. U. (1994). Resetting the biological clock: mediation of nocturnal circadian shifts by glutamate and NO. *Science* 266 1713–1717. 10.1126/science.7527589 7527589

[B18] Ding J. M. Faiman L. E. Hurst W. J. Kuriashkina L. R. Gillette M. U. (1997). Resetting the biological clock: mediation of nocturnal CREB phosphorylation via light, glutamate, and nitric oxide. *J. Neurosci.* 17 667–675. 10.1523/jneurosci.17-02-00667.1997 8987789 PMC6573241

[B19] Emery A. Eiden M. V. Mustafa T. Eiden L. E. (2013). GPCR-Gs signaling to ERK is controlled by the cAMP-sensing guanine nucleotide exchange factor NCS/Rapgef2 in neuronal and endocrine cells. *Sci. Signal.* 6:ra51 10.1126/scisignal.2003993 PMC3932028 23800469

[B20] Emery A. C. Eiden L. E. (2012). Signaling through the neuropeptide GPCR PAC1 induces neuritogenesis via a single linear cAMP- and ERK-dependent pathway using a novel cAMP sensor. *FASEB J.* 26 3199–3211. 10.1096/fj.11-203042 22532442 PMC3405272

[B21] Frödin M. Peraldi P. Van Obberghen E. (1994). Cyclic AMP activates the mitogen-activated protein kinase cascade in PC12 cells. *J. Biol. Chem.* 269 6207–6214. 7907091

[B22] Gerdin M. J. Eiden L. E. (2007). Regulation of PC12 cell differentiation by cAMP signaling to ERK independent of PKA: do all the connections add up? *Sci. STKE* 2007:15. 17440132 10.1126/stke.3822007pe15 PMC4183209

[B23] Gillette M. U. (1986). The suprachiasmatic nuclei: circadian phase-shifts induced at the time of hypothalamic slice preparation are preserved in vitro. *Brain Res.* 379 176–181. 10.1016/0006-8993(86)90273-8 3742212

[B24] Gillette M. U. Mitchell J. W. (2002). Signaling in the suprachiasmatic nucleus: selectively responsive and integrative. *Cell Tissue Res.* 309 99–107. 10.1007/s00441-002-0576-1 12111540

[B25] Gillette M. U. Prosser R. A. (1988). Circadian rhythm of the rat suprachiasmatic brain slice is rapidly reset by daytime application of cAMP analogs. *Brain Res.* 474 348–352. 10.1016/0006-8993(88)90449-0 2850092

[B26] Ginty D. D. Kornhauser J. M. Thompson M. A. Bading H. Mayo K. E. Takahashi J. S. (1993). Regulation of CREB phosphorylation in the suprachiasmatic nucleus by light and a circadian clock. *Science* 260 238–241. 10.1126/science.8097062 8097062

[B27] Green D. J. Gillette R. (1982). Circadian rhythm of firing rate recorded from single cells in the rat suprachiasmatic brain slice. *Brain Res.* 245 198–200. 10.1016/0006-8993(82)90361-4 6889453

[B28] Hamelink C. Tjurmina O. Damadzic R. Young W. S. Weihe E. Lee H. W. (2002). Pituitary adenylate cyclase-activating polypeptide is a sympathoadrenal neurotransmitter involved in catecholamine regulation and glucohomeostasis. *Proc. Natl. Acad. Sci. U.S.A.* 99 461–466. 10.1073/pnas.012608999 11756684 PMC117582

[B29] Hammack S. E. May V. (2015). Pituitary adenylate cyclase activating polypeptide in stress-related disorders: data convergence from animal and human studies. *Biol. Psychiatry* 78 167–177. 10.1016/j.biopsych.2014.12.003 25636177 PMC4461555

[B30] Hannibal J. Ding J. M. Chen D. Fahrenkrug J. Larsen P. J. Gillette M. U. (1997). Pituitary adenylate cyclase-activating peptide (PACAP) in the retinohypothalamic tract: a potential daytime regulator of the biological clock. *J. Neurosci.* 17 2637–2644. 10.1523/jneurosci.17-07-02637.1997 9065523 PMC6573509

[B31] Hannibal J. Ding J. M. Chen D. Fahrenkrug J. Larsen P. J. Gillette M. U. (1998). Pituitary adenylate cyclase activating peptide (PACAP) in the retinohypothalamic tract: a daytime regulator of the biological clock. *Ann. N. Y. Acad. Sci.* 865 197–206. 10.1111/j.1749-6632.1998.tb11179.x 9928013

[B32] Hannibal J. Fahrenkrug J. (2004). Target areas innervated by PACAP-immunoreactive retinal ganglion cells. *Cell Tissue Res.* 316 99–113. 10.1007/s00441-004-0858-x 14991397

[B33] Hannibal J. Hindersson P. Knudsen S. M. Georg B. Fahrenkrug J. (2002). The photopigment melanopsin is exclusively present in pituitary adenylate cyclase-activating polypeptide-containing retinal ganglion cells of the retinohypothalamic tract. *J. Neurosci.* 22:RC191. 11756521 10.1523/JNEUROSCI.22-01-j0002.2002 PMC6757615

[B34] Hannibal J. Jamen F. Nielsen H. S. Journot L. Brabet P. Fahrenkrug J. (2001a). Dissociation between light-induced phase shift of the circadian rhythm and clock gene expression in mice lacking the pituitary adenylate cyclase activating polypeptide type 1 receptor. *J. Neurosci.* 21 4883–4890. 10.1523/jneurosci.21-13-04883.2001 11425915 PMC6762353

[B35] Hannibal J. Vrang N. Card J. P. Fahrenkrug J. (2001b). Light-dependent induction of cFos during subjective day and night in PACAP-containing ganglion cells of the retinohypothalamic tract. *J. Biol. Rhythms* 16 457–470. 10.1177/074873001129002132 11669419

[B36] Hannibal J. Moller M. Ottersen O. P. Fahrenkrug J. (2000). PACAP and glutamate are co-stored in the retinohypothalamic tract. *J. Comp. Neurol.* 418 147–155. 10.1002/(sici)1096-9861(20000306)418:2<147::aid-cne2>3.0.co;2-# 10701440

[B37] Harmar A. J. Marston H. M. Shen S. Spratt C. West K. M. Sheward W. J. (2002). The VPAC2 receptor is essential for circadian function in the mouse suprachiasmatic nuclei. *Cell* 109 497–508. 10.1016/s0092-8674(02)00736-5 12086606

[B38] Harrington M. E. Hoque S. Hall A. Golombek D. Biello S. (1999). Pituitary adenylate cyclase activating peptide phase shifts circadian rhythms in a manner similar to light. *J. Neurosci.* 19 6637–6642. 10.1523/jneurosci.19-15-06637.1999 10414992 PMC6782812

[B39] Hashimoto H. Kunugi A. Arakawa N. Shintani N. Fujita T. Kasai A. (2003). Possible involvement of a cyclic AMP-dependent mechanism in PACAP-induced proliferation and ERK activation in astrocytes. *Biochem. Biophys. Res. Commun.* 311 337–343. 10.1016/j.bbrc.2003.10.005 14592419

[B40] Kawaguchi C. Tanaka K. Isojima Y. Shintani N. Hashimoto H. Baba A. (2003). Changes in light-induced phase shift of circadian rhythm in mice lacking PACAP. *Biochem. Biophys. Res. Commun.* 310 169–175. 10.1016/j.bbrc.2003.09.004 14511666

[B41] Keenan W. T. Rupp A. C. Ross R. A. Somasundaram P. Hiriyanna S. Wu Z. (2016). A visual circuit uses complementary mechanisms to support transient and sustained pupil constriction. *eLife* 5:e15392. 10.7554/eLife.15392 27669145 PMC5079752

[B42] Macdonald D. S. Weerapura M. Beazely M. A. Martin L. Czerwinski W. Roder J. C. (2005). Modulation of NMDA receptors by pituitary adenylate cyclase activating peptide in CA1 neurons requires G alpha q, protein kinase C, and activation of Src. *J. Neurosci.* 25 11374–11384. 10.1523/jneurosci.3871-05.2005 16339032 PMC6725893

[B43] MacDonald J. F. Jackson M. F. Beazely M. A. (2007). G protein-coupled receptors control NMDARs and metaplasticity in the hippocampus. *Biochim. Biophys. Acta* 1768 941–951. 10.1016/j.bbamem.2006.12.006 17261268

[B44] May V. Buttolph T. R. Girard B. M. Clason T. A. Parsons R. L. (2014). PACAP-induced ERK activation in HEK cells expressing PAC1 receptors involves both receptor internalization and PKC signaling. *Am. J. Physiol. Cell Physiol.* 306 C1068–C1079. 10.1152/ajpcell.00001.2014 24696141 PMC4042093

[B45] McNulty S. Schurov I. L. Sloper P. J. Hastings M. H. (1998). Stimuli which entrain the circadian clock of the neonatal Syrian hamster *in vivo* regulate the phosphorylation of the transcription factor CREB in the suprachiasmatic nucleus *in vitro*. *Eur. J. Neurosci.* 10 1063–1072. 10.1046/j.1460-9568.1998.00114.x 9753174

[B46] Michel S. Itri J. Han J. H. Gniotczynski K. Colwell C. S. (2006). Regulation of glutamatergic signalling by PACAP in the mammalian suprachiasmatic nucleus. *BMC Neurosci.* 7:15. 10.1186/1471-2202-7-15 16483357 PMC1388226

[B47] Miles O. Thrailkill E. A. Linden A. K. May V. Bouton M. E. Hammack S. E. (2016). *Cocaine self-Administration Alters Endogenous Pituitary Adenylate Cyclase Activating Peptide (PACAP) Levels in the Bed Nucleus of the Stria Terminalis (BNST).* San Diego, CA: Society for Neuroscience.

[B48] Miles O. W. Thrailkill E. A. Linden A. K. May V. Bouton M. E. Hammack S. E. (2017). Pituitary adenylate cyclase-activating peptide in the bed nucleus of the stria terminalis mediates stress-induced reinstatement of cocaine seeking in rats. *Neuropsychopharmacology* 43 978–986. 10.1038/npp.2017.135 28656976 PMC5854788

[B49] Missig G. Mei L. Vizzard M. A. Braas K. M. Waschek J. A. Ressler K. J. (2015). Parabrachial PACAP activation of amygdala endosomal ERK signaling regulates the emotional component of pain. *Biol. Psychiatr.* 81 671–682. 10.1016/j.biopsych.2016.08.025 28057459 PMC5332340

[B50] Missig G. Roman C. W. Vizzard M. A. Braas K. M. Hammack S. E. May V. (2014). Parabrachial nucleus (PBn) pituitary adenylate cyclase activating polypeptide (PACAP) signaling in the amygdala: implication for the sensory and behavioral effects of pain. *Neuropharmacology* 86 38–48. 10.1016/j.neuropharm.2014.06.022 24998751 PMC4322675

[B51] Miyata A. Arimura A. Dahl R. R. Minamino N. Uehara A. Jiang L. (1989). Isolation of a novel 38 residue-hypothalamic polypeptide which stimulates adenylate cyclase in pituitary cells. *Biochem. Biophys. Res. Commun.* 164 567–574. 10.1016/0006-291x(89)91757-9 2803320

[B52] Okazaki K. Itoh Y. Ogi K. Ohkubo S. Onda H. (1995). Characterization of murine PACAP mRNA. *Peptides* 16 1295–1299. 10.1016/0196-9781(95)02018-r 8545254

[B53] Panda S. Sato T. K. Castrucci A. M. Rollag M. D. DeGrip W. J. Hogenesch J. B. (2002). Melanopsin (Opn4) requirement for normal light-induced circadian phase shifting. *Science* 298 2213–2216. 10.1126/science.1076848 12481141

[B54] Piggins H. D. Marchant E. G. Goguen D. Rusak B. (2001). Phase-shifting effects of pituitary adenylate cyclase activating polypeptide on hamster wheel-running rhythms. *Neurosci. Lett.* 305 25–28. 10.1016/s0304-3940(01)01796-7 11356299

[B55] Prosser R. A. Gillette M. U. (1989). The mammalian circadian clock in the suprachiasmatic nuclei is reset in vitro by cAMP. *J. Neurosci.* 9 1073–1081. 10.1523/jneurosci.09-03-01073.1989 2538580 PMC6569955

[B56] Prosser R. A. Gillette M. U. (1991). Cyclic changes in cAMP concentration and phosphodiesterase activity in a mammalian circadian clock studied in vitro. *Brain Res.* 568 185–192. 10.1016/0006-8993(91)91396-i 1667616

[B57] Prosser R. A. McArthur A. J. Gillette M. U. (1989). cGMP induces phase shifts of a mammalian circadian pacemaker at night, in antiphase to cAMP effects. *Proc. Natl. Acad. Sci. U.S.A.* 86 6812–6815. 10.1073/pnas.86.17.6812 2549549 PMC297936

[B58] Ravni A. Vaudry D. Gerdin M. J. Eiden M. V. Falluel-Morel A. Gonzalez B. (2008). A cAMP-dependent, PKA-independent signaling pathway mediating neuritogenesis through Egr1 in PC12 cells. *Mol. Pharmacol.* 73 1688–1708. 10.1124/mol.107.044792 18362103 PMC4188547

[B59] Reimer M. Moller K. Sundler F. Hannibal J. Fahrenkrug J. Kanje M. (1999). Increased expression, axonal transport and release of pituitary adenylate cyclase-activating polypeptide in the cultured rat vagus nerve. *Neuroscience* 88 213–222. 10.1016/s0306-4522(98)00240-1 10051202

[B60] Ruby N. F. Brennan T. J. Xie X. Cao V. Franken P. Heller H. C. (2002). Role of melanopsin in circadian responses to light. *Science* 298 2211–2213. 10.1126/science.1076701 12481140

[B61] Schafer M. K. Mahata S. K. Stroth N. Eiden L. E. Weihe E. (2010). Cellular distribution of chromogranin a in excitatory, inhibitory, aminergic and peptidergic neurons of the rodent central nervous system. *Regul. Pept.* 165 36–44. 10.1016/j.regpep.2009.11.021 20005907 PMC2997928

[B62] Schmidt S. D. Myskiw J. C. Furini C. R. Schmidt B. E. Cavalcante L. E. Izquierdo I. (2015). PACAP modulates the consolidation and extinction of the contextual fear conditioning through NMDA receptors. *Neurobiol. Learn. Mem.* 118C 120–124. 10.1016/j.nlm.2014.11.014 25490058

[B63] Shen S. Spratt C. Sheward W. J. Kallo I. West K. Morrison C. F. (2000). Overexpression of the human VPAC2 receptor in the suprachiasmatic nucleus alters the circadian phenotype of mice. *Proc. Natl. Acad. Sci. U.S.A.* 97 11575–11580. 10.1073/pnas.97.21.11575 11027354 PMC17242

[B64] Shibata S. Oomura Y. Kita H. Hattori K. (1982). Circadian rhythmic changes of neuronal activity in the suprachiasmatic nucleus of the rat hypothalamic slice. *Brain Res.* 247 154–158. 10.1016/0006-8993(82)91041-1 7127113

[B65] Tischkau S. A. Gallman E. A. Buchanan G. F. Gillette M. U. (2000). Differential cAMP gating of glutamatergic signaling regulates long-term state changes in the suprachiasmatic circadian clock. *J. Neurosci.* 20 7830–7837. 10.1523/jneurosci.20-20-07830.2000 11027248 PMC6772885

[B66] Tischkau S. A. Mitchell J. W. Tyan S. H. Buchanan G. F. Gillette M. U. (2003). Ca2+/cAMP response element-binding protein (CREB)-dependent activation of Per1 is required for light-induced signaling in the suprachiasmatic nucleus circadian clock. *J. Biol. Chem.* 278 718–723. 10.1074/jbc.m209241200 12409294

[B67] Vaudry D. Gonzalez B. J. Basille M. Yon L. Fournier A. Vaudry H. (2000). Pituitary adenylate cyclase-activating polypeptide and its receptors: from structure to functions. *Pharmacol. Rev.* 52 269–324. 10835102

[B68] von Gall C. Duffield G. E. Hastings M. H. Kopp M. D. Dehghani F. Korf H. W. (1998). “CREB in the mouse SCN: a molecular interface coding the phase-adjusting stimuli light, glutamate, PACAP, and melatonin for clockwork access. *J. Neurosci.* 18 10389–10397. 10.1523/jneurosci.18-24-10389.1998 9852576 PMC6793329

[B69] Zhang L. Hernández V. S. Weihe E. Eiden L. E. Jiang S. Z. Lindberg P. T. (2018). *Conservation of Retinohypothalamic, Hippocampal, and Amygdalar PACAPergic Circuits in Mouse and Rat.* San Diego, CA: Society for Neuroscience Annual Meeting Available at: https://www.sfn.org/meetings/neuroscience-2018/abstracts/neuroscience-2018-abstracts

